# Empirical-Null Calibration Challenges Comparative CNS Safety Signals for Androgen-Receptor Pathway Inhibitors: A FAERS Re-Analysis

**DOI:** 10.3390/ph19081213

**Published:** 2026-08-01

**Authors:** Abdurrahman İnkaya, Hasan Samet Güngör

**Affiliations:** Department of Urology, Ümraniye Training and Research Hospital, University of Health Sciences, İstanbul 34764, Türkiye

**Keywords:** prostate cancer, androgen-receptor pathway inhibitors, pharmacovigilance, disproportionality, empirical calibration, falls, fractures, bone health, agent selection, FAERS

## Abstract

**Background/Objectives:** The four androgen-receptor pathway inhibitors (ARPIs) for advanced prostate cancer are broadly comparable in efficacy, so the choice between them depends on tolerability and, in elderly patients, on central nervous system (CNS) safety. CNS signals from the FDA Adverse Event Reporting System (FAERS) now inform that choice, yet they rest on uncalibrated disproportionality, which cannot separate pharmacology from reporting. **Methods:** Across the full FAERS database (20,328,575 reports), we computed reporting odds ratios (ROR) for enzalutamide, apalutamide, darolutamide, and abiraterone over 32 drug–event pairs, recalibrated every estimate against a drug-specific empirical null from 18 negative controls, and corrected for multiplicity. The null was fitted two ways and validated against twelve label-established positive controls. **Results:** Uncalibrated analysis flagged 22 of 32 pairs, and also 41 of 72 control pairs on events judged to carry no true effect, so its null does not hold. Calibration removes that bias, but the verdict then depends on how the null is fitted: with its dispersion taken as the sample standard deviation of the control estimates, no pair reached q < 0.05, while under the maximum-likelihood fit the framework prescribes six did, three of them being CNS pairs. Positive controls confirmed that calibration retains genuine agent-specific effects, and the detection floor lay above every CNS estimate for the same agent. **Conclusions:** Comparative ARPI CNS signals in FAERS are not identifiable; the verdict is set by the calibration design rather than by the drugs, so such signals should not stand alone in agent selection. The firmer safety axis remains fall and fracture risk, the class’s dominant serious harm in trials and guidelines.

## 1. Introduction

Androgen-receptor pathway inhibitors (ARPIs) have transformed advanced prostate cancer. Four agents dominate practice. Enzalutamide, apalutamide, and darolutamide antagonize the androgen receptor; abiraterone acetate inhibits CYP17A1. Within first-line metastatic castration-sensitive disease, indirect comparison puts their efficacy broadly on a par [1], so within a given indication, tolerability decides between them. In an elderly and often frail population, central nervous system (CNS) safety carries much of that decision, alongside cardiovascular and metabolic tolerability. Cognitive complaints, fatigue, and fear of decline now weigh heavily in that choice.

The four agents differ in one property that matters here: how well they cross the blood–brain barrier (BBB). Enzalutamide and apalutamide cross readily. Darolutamide was built for low penetrance, and abiraterone has little direct central activity. A randomized crossover study found differential cerebral blood flow after enzalutamide and darolutamide, consistent with darolutamide’s lower blood–brain barrier penetration [2]. That gradient now reaches the bedside, where darolutamide is preferred on cognitive grounds. The harm with the firmest evidence, though, is not cognitive. Randomized-trial meta-analyses show that ARPIs added to androgen-deprivation therapy roughly double the risk of falls and fractures [3,4], while a network meta-analysis found that each agent carries a distinct central nervous system profile, with darolutamide free of significant CNS associations apart from fatigue [5].

This is where the real-world evidence enters, and where it fails. Several analyses of the FDA Adverse Event Reporting System (FAERS) have compared ARPIs, for cardiovascular safety [6] and for broader multi-system safety [7]; the broader analysis reported prominent nervous system signals for the AR antagonists. That signal feeds the cognitive-safety narrative that guides selection. They rest on uncalibrated disproportionality, which declares a signal against a theoretical null of no association. That null breaks down when co-indicated drugs are compared. A newer agent under intensive surveillance accumulates reports faster, and report volume climbs with exposure and time on the market—the Weber effect [8], a naïve comparison then measures the drug and its reporting habits at once.

Empirical-null calibration is well established in observational effect estimation [9,10], though its transfer to spontaneous-report data is recent and not yet routine practice. A set of outcomes with no plausible true effect measures a data stream’s own bias and its residual uncertainty, and each estimate is recalibrated against that null. To our knowledge, it has not been applied to a comparison of ARPIs. We asked a deliberately conservative question. Once the reporting bias is removed and the 32 tested pairs are corrected for multiplicity, does any comparative ARPI CNS signal stay strong enough to justify choosing one agent over another, and, if not, which axis should guide that choice?

Spontaneous reports cannot prove causation. The question here is narrower: whether the CNS signals now used in selection survive calibration at all.

## 2. Results

### 2.1. Cohort

Report volumes differed sharply by agent. Enzalutamide contributed 58,003 reports, abiraterone 40,471, apalutamide 11,281, and darolutamide 5120, against a FAERS background of 20,328,575.

The cases were almost entirely male and elderly, as expected for advanced prostate cancer. Sex was recorded in 84–96% of reports and was male in 99.2–99.6% of those across agents; age was recorded in 51–60% and gave a median of 74–76 years, with an interquartile range spanning 66–82 (Supplementary Table S1). Female cases were rare, 0.4–0.7% of reports with sex recorded, likely reflecting off-label androgen-receptor-positive breast cancer use of enzalutamide and abiraterone, accidental caregiver exposure, and sex-field miscoding; at that share they do not affect the disproportionality estimates, which were not sex-stratified.

### 2.2. Naïve Disproportionality and Negative Controls

**Figure 1:**
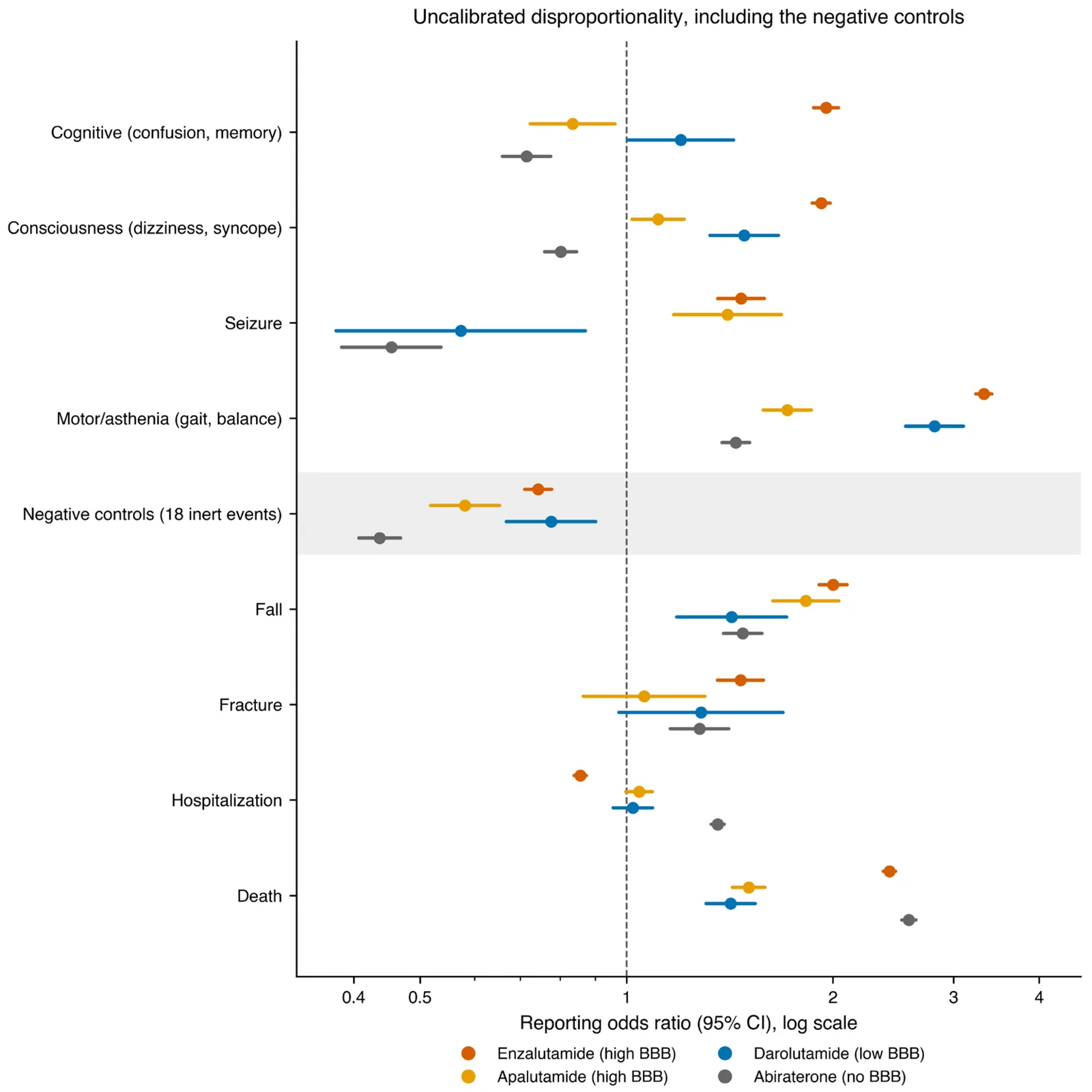
Raw reporting odds ratios (RORs) with 95% confidence intervals (CIs) for the four androgen-receptor pathway inhibitors (ARPIs) across event groups, including the pooled 18 decontaminated negative controls. Forty-one of the 72 drug–control pairs reach nominal significance although the events were selected to carry no true effect, and 34 of those lie below one: the naive null is wrong, and these agents under-report inert events rather than over-report them.

Uncalibrated disproportionality flagged 22 of 32 drug–event pairs (Figure 1). It flagged the negative controls too. Of the 72 drug–control pairs, 41 reached nominal significance on events selected to carry no true effect, which by itself shows that the theoretical null is wrong for this comparison. The direction is instructive: 34 of those 41 were significant below one rather than above it. Against the whole database, these agents under-report banal events rather than over-report them, which is what an oncology reporting stream would be expected to do. A naïve signal for these drugs therefore cannot tell pharmacology from reporting, the same flaw in the earlier uncalibrated FAERS analyses of this class [6,7]. That reading rests on the assumption that the control events are genuinely inert, which is a mechanistic judgment rather than an established fact, and it is the assumption the whole design depends on.

Pooled into one 2 × 2 table, the 18 inert events gave reporting odds ratios of 0.74 for enzalutamide, 0.58 for apalutamide, 0.78 for darolutamide, and 0.44 for abiraterone, close to the unweighted means that feed the calibration (exp(μ) of 0.75, 0.58, 0.88, and 0.48). On a clean control set, the two summaries agree. They diverge only when the set is contaminated: the initial twelve-event set, which still held five on-label effects, pooled to 1.86, 2.24, 2.18, and 1.01, above one for all three AR antagonists (Supplementary Figure S1d). That divergence is a diagnostic of contamination rather than of skewed control counts, and it is what prompted the rebuild described in Section 4.2. We report both sets so the distinction stays visible.

### 2.3. Signals Fall to Calibration, and How Far They Fall Depends on the Null

**Figure 2:**
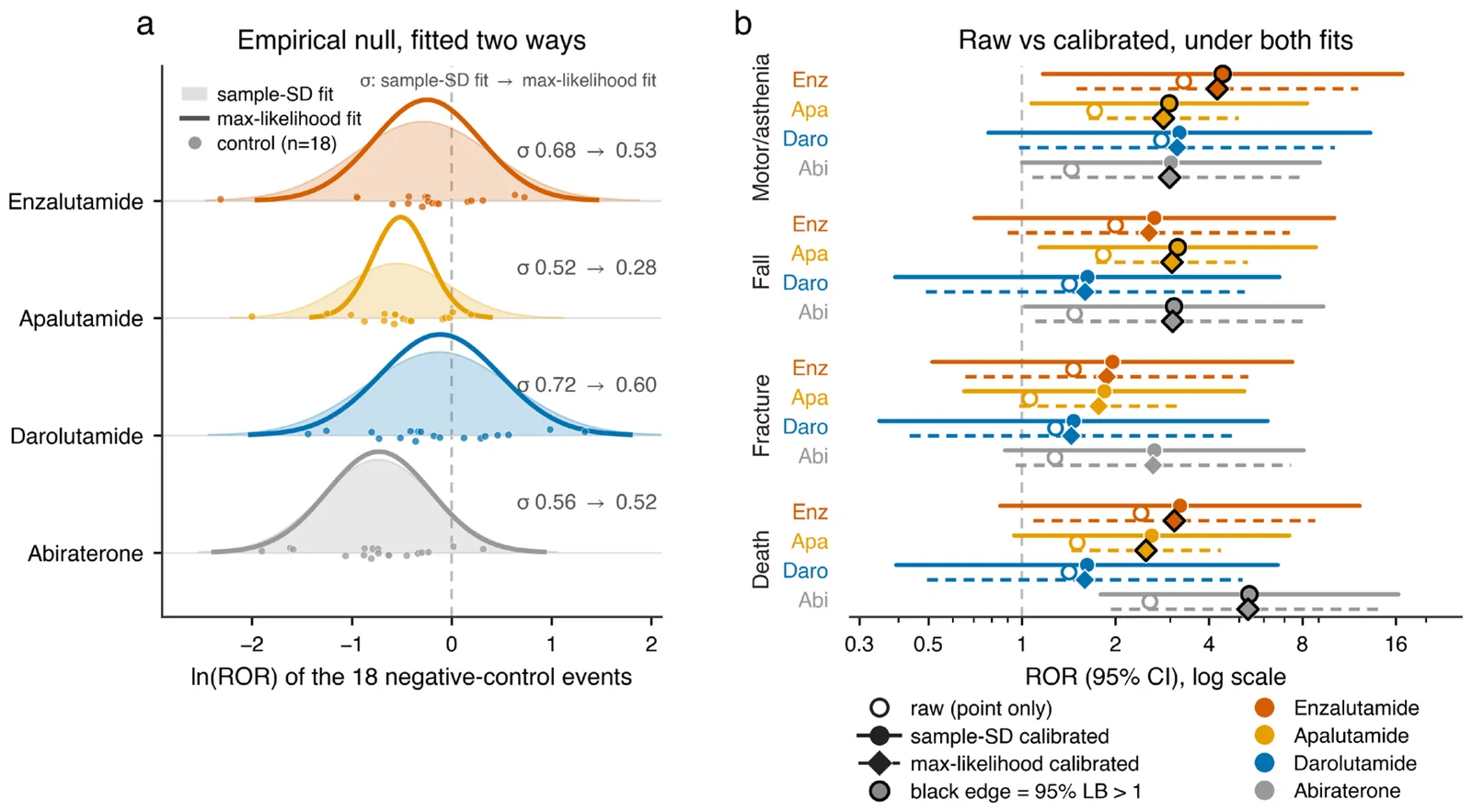
The empirical null, fitted two ways. (**a**) For each agent, the ln(ROR) of the 18 negative-control events (dots), with the null fitted as the sample standard deviation of those values (shaded) and by the maximum likelihood the framework prescribes (solid line). Both densities are area-normalized, so the narrower fit is taller and ends sooner, and each row is annotated with the two σ values. The maximum-likelihood fit is narrower because it separates the controls’ own sampling error from the systematic spread. The gray dashed line marks ln(ROR) = 0. (**b**) Raw estimates (open rings, point only) against calibrated estimates for four event groups, with the drugs labeled by colored initial. Filled circles with solid intervals are the sample-standard-deviation fit; filled diamonds with dashed intervals are the maximum-likelihood fit. A black marker edge marks a calibrated 95% lower bound above one, which is not the same as surviving multiplicity correction.

The decontaminated controls gave drug-specific nulls with negative means (μ: enzalutamide −0.29, apalutamide −0.55, darolutamide −0.13, abiraterone −0.74) and a wide spread (σ 0.52–0.72), which confirms that the nominal intervals were too narrow (Figure 2a). Calibration left five of the 22 raw signals with a 95% lower bound above one (Table 1, Figure 2b). Under this primary mechanism-filtered null, multiplicity correction took the rest: none of the 32 calibrated *p*-values reached significance, the smallest q being 0.088, for abiraterone death, with the other four at q = 0.26 (Supplementary Figure S1a; the full 32-pair raw and calibrated estimates are in Supplementary Table S2). Whether that verdict holds under the framework’s own fit of the null is taken up in the next paragraph, and across other defensible control sets in Section 2.7. Under this fit, the broad cognitive and consciousness signals, the ones most of the prior literature leans on, were already gone at the calibration step, before multiplicity was applied.

The multiplicity verdict is not a property of the data alone. Under the sample-standard-deviation fit, no pair reached q < 0.05, the smallest being 0.088. Under the maximum-likelihood fit the framework prescribes, the fitted spread is narrower (σ 0.28 to 0.60 rather than 0.52 to 0.72) because the controls’ own sampling error is no longer counted as systematic spread; eleven pairs then kept a calibrated lower bound above one and six reached q < 0.05, the smallest being 0.003. Three of the six are CNS pairs: apalutamide with motor/asthenia and with seizure, and enzalutamide with motor/asthenia. Restricting the controls to those with at least three or at least five reports, still under the maximum-likelihood fit, leaves that result unchanged. Two defensible but non-equivalent implementations of one framework thus give opposite answers on the same data, and that is the central result of this study.

**Figure 3:**
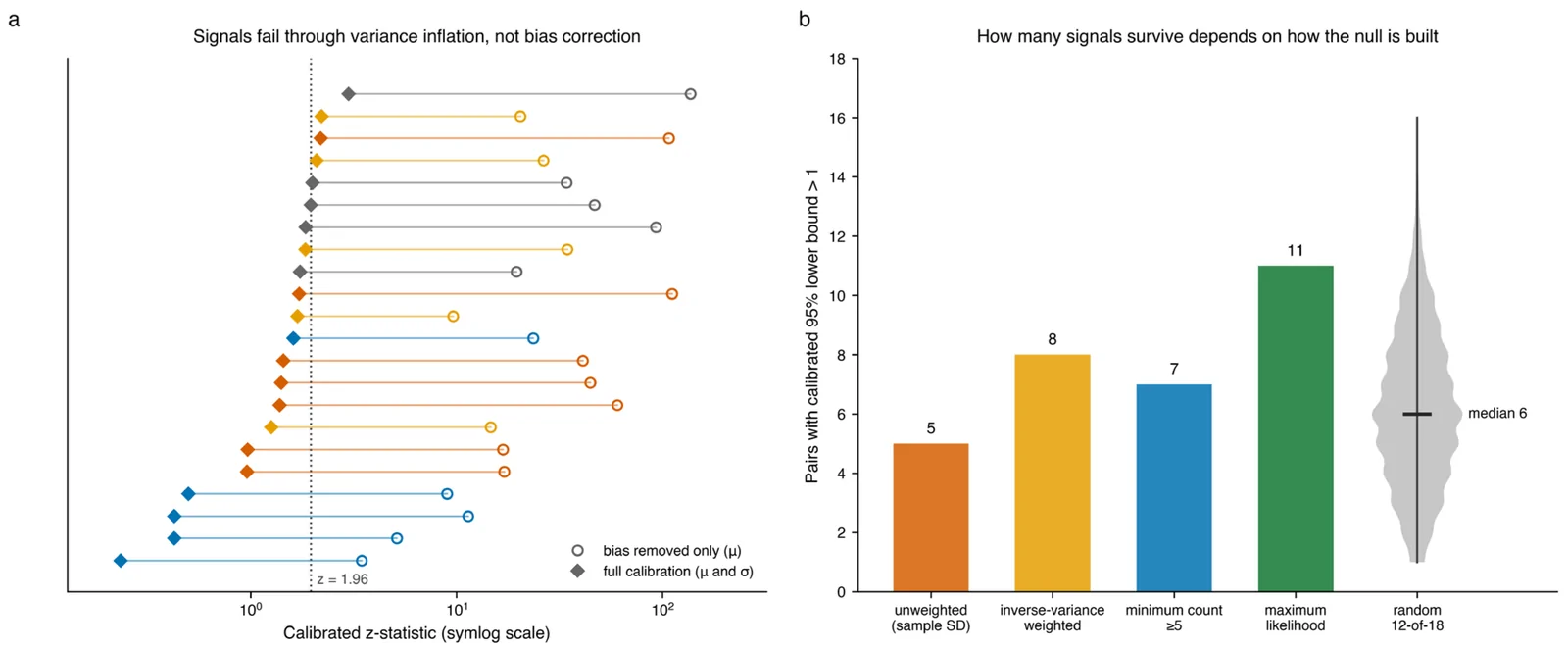
Signal loss and its dependence on how the null is built. (**a**) For each raw-significant pair, the calibrated z after removing bias only (μ; open circles) against full calibration (μ and σ; filled diamonds). Removing bias alone leaves all 22 significant; significance is lost only when the residual variance σ is added, so signals fail through variance inflation rather than bias correction. (**b**) Pairs with a calibrated 95% lower bound above one under four full-set null estimators and across 10,000 random 12-of-18 negative-control subsets (violin). The resampled count runs from one to 16, so no individual surviving pair is treated as established.

Why the signals fell matters as much as that they fell. Take each raw signal and remove only the bias term, μ, which is negative for all four drugs: every one stays significant. Significance goes only when the residual variance σ is added to the standard error (Figure 3a). Because the bias term is negative for every drug, removing it can only strengthen a raw signal, never explain a loss; this is arithmetic, not a mechanism. What removes them is the unmodelled spread between events, once that spread is carried through to the estimate. This is a statement about robustness under honest uncertainty, not a verdict of artifact.

The number of survivors depended on how the null was built (Figure 3b). The primary unweighted fit left five; an inverse-variance-weighted null left eight; a minimum-count subset left seven; the maximum-likelihood fit left 11. Across 10,000 random 12-of-18 control subsets, the count ran a median of six (interquartile range [IQR] 4–8, range 1–16). The direction never changed for calibration: few signals survive it. Multiplicity is the step that needs care—under the primary null none survives, but that verdict is not stable to the null, as Section 2.7 shows. The identity of the survivors also shifted across designs, which is why no single surviving pair is treated as real.

### 2.4. Validation: What Calibration Keeps, and What It Cannot See

Calibration left the established effects intact. Of the 48 drug–event pairs formed from twelve label-established positive-control events, 32 were significant before calibration; 13 kept a calibrated lower bound above one under the sample-standard-deviation fit, nine of them at q < 0.05, 20 under the maximum-likelihood fit, and 18 of them at q < 0.05. The survivors are the effects with the firmest label basis and the clearest drug specificity (Table 2). Hot flush, an androgen-axis class effect, survived for all four agents with calibrated RORs of 22.0 to 40.7. Hypokalaemia, the mineralocorticoid-excess effect specific to abiraterone, gave a calibrated ROR of 17.98 (95% CI 5.95–54.31) for abiraterone against 0.61 to 1.54 for the other three agents; hypothyroidism and rash, both apalutamide effects, behaved the same way. The procedure therefore still separates agents when a real between-agent difference exists, which is the property a negative comparative finding needs.

It separates them only above a floor. The smallest calibrated ROR detectable with 80% power at a two-sided α of 0.05 was 4.3 for apalutamide, 4.9 for abiraterone, 6.7 for enzalutamide, and 7.6 for darolutamide under the sample-standard-deviation fit. Every calibrated CNS estimate in this study lay between 0.65 and 4.43, below the floor for its own agent. The positive controls make the same point empirically: no positive control with a calibrated ROR under 4.7 reached q < 0.05, and gynaecomastia for enzalutamide, a genuine androgen-axis effect, did not survive. A CNS difference in the magnitude at issue would not have been detected by this design had it been present, which is why we read the comparative result as unresolved rather than as negative.

### 2.5. What Survival Does Not Show: The BBB Gradient and Single Controls

The BBB story does not survive a second look (Supplementary Figure S1, Figure 2b). On raw disproportionality darolutamide, the low-penetrance agent had a higher motor/asthenia ROR (2.81) than apalutamide (1.72). It failed calibration not because its signal was weaker but because it is the smallest dataset, at 5120 reports. The small dataset makes the noisiest 18-point null and the widest σ (0.72), and so the widest calibrated interval. Differential survival across agents tracks how precisely each null is measured and which follows report count, as much as it tracks pharmacology. The BBB gradient cannot be read from these data.

No single control drove the null. Across the 18 leave-one-out fits, μ shifted within about ±0.12 on the ln scale for every drug, and no one control flipped the result (Supplementary Figure S1b). The most influential single control, conjunctivitis for enzalutamide with five reports, moved μ from −0.29 to −0.17, short of rescuing any signal. What is decisive is the control set as a whole, not any one event.

### 2.6. Time-to-Onset and the Fall–Fracture Structure

Onset timing differed by agent but carried no BBB order. Median motor/asthenia onset was earliest for darolutamide (92 days) and latest for abiraterone (266 days), with enzalutamide (141 days) and apalutamide (126 days) between them. The enzalutamide Weibull shape exceeded 1 (k = 1.28), a hazard rising over the first year (Supplementary Figure S2). Because FAERS records no reaction-level date, this is exploratory and kept in the Supplementary Materials; it carries no pharmacokinetic reading.

**Figure 4:**
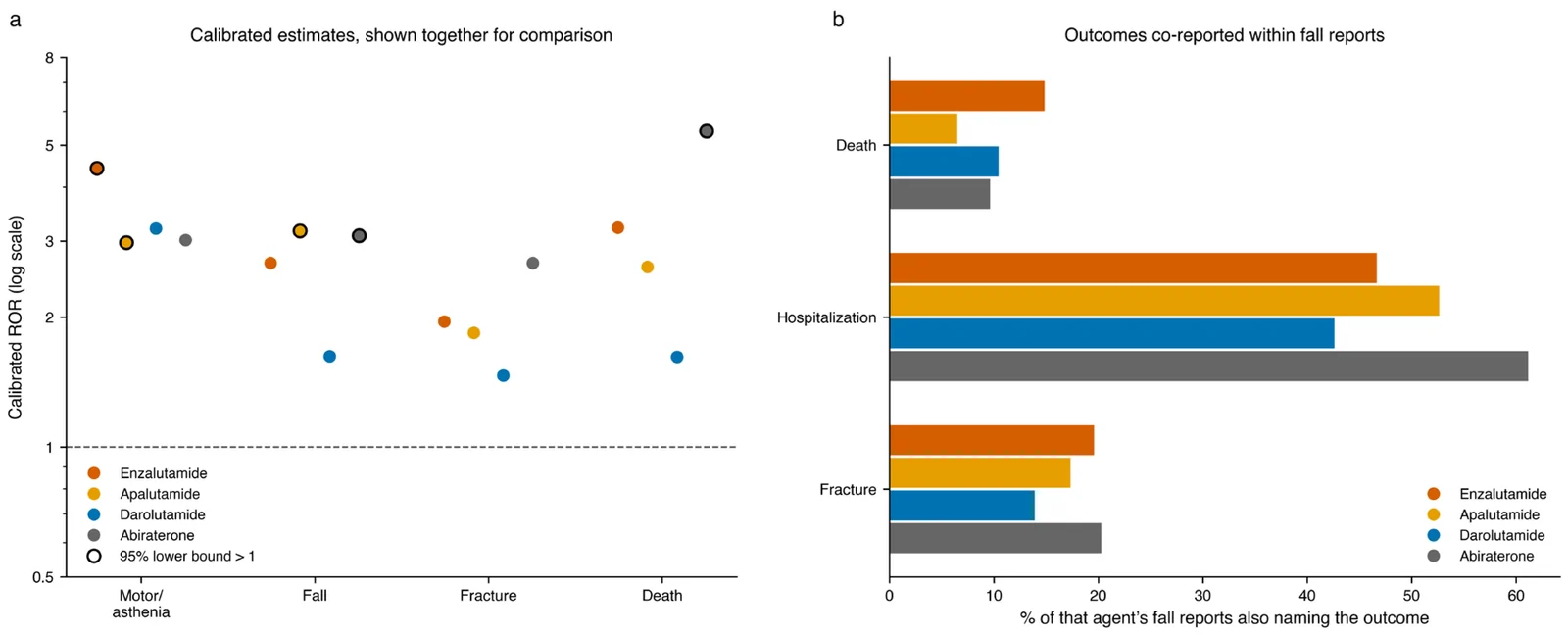
Within-report fall-centered co-reporting structure. (**a**) Calibrated ROR for motor/asthenia, fall, fracture, and death, shown together for comparison and not as a demonstrated temporal sequence; a black marker edge marks a calibrated 95% lower bound above one. (**b**) Percentage of each agent’s fall reports that also name a fracture, a hospitalization, or a death.

One pattern held up on its own terms: falls and fractures traveled together within reports (Figure 4, Supplementary Figure S1c). A fracture appeared in 13.9–20.3% of fall reports, against 0.6–1.6% of non-fall serious reports for the same drug. That is a 10- to 38-fold enrichment in the odds, significant for every agent (Fisher’s exact *p* < 10^−6^). The gap is far wider than generic co-reporting of a serious event would give. It is also close to expected: a fall that breaks a bone is entered as one event, and injurious falls are the ones most often reported, so the enrichment reflects how reports are generated at least as much as any biology. Its size is therefore not the informative part; what it marks is the presence of the fall–fracture pairing in the reporting stream. This is co-reporting, read strictly. FAERS captures co-mention inside one report, not a sequence in time, so nothing here says the fall came first or caused the fracture. Falls were also co-reported with hospitalization in 42.6–61.1% and with death in 6.5–14.9%; those have no clean reference class and are given only as description. The per-drug fall counts, fracture-within-fall counts, and Fisher exact *p* for each agent are listed in Supplementary Table S4.

### 2.7. Sensitivity Analyses

Restricting to suspect-drug reports kept about 99% of the data and left the calibrated estimates essentially where they were. The contaminated and decontaminated control sets, by contrast, told different stories. The contaminated set inflated the null so far that all 22 raw signals vanished, which again shows that control selection governs this design. The multiplicity verdict was itself examined the same way. Under the primary unweighted null, no pair reached q < 0.05 (minimum q = 0.088), but that verdict was not stable to the null: across 10,000 random 12-of-18 control subsets, 45.4% of draws produced at least one pair at q < 0.05 (median minimum q = 0.058, IQR 0.020–0.123), and an inverse-variance-weighted null left four pairs at q < 0.05 (minimum q = 0.028); the minimum-count subset left none (Supplementary Figure S3). The honest reading is therefore not that no signal survives multiplicity, but that under most calibration designs none does, while a minority of equally defensible designs preserve a small number.

The same instability shows when the control set itself is resampled. Across 10,000 bootstrap resamples of the 18 controls, at least one pair reached q < 0.05 in 61.5% of draws under the sample-standard-deviation fit and in 98.9% under the maximum-likelihood fit. Thinning the control set to 12 of 18 lowers survival while a full-size bootstrap raises it, but a verdict of no surviving signal is a minority outcome even under the more conservative of the two fits, which is the clearest statement we can make of how far this design determines its own answer.

openFDA offers no fully deduplicated case build, so a duplicate-flag analysis was run. Dropping every report with openFDA’s duplicate flag removed 26–81% of reports per drug, yet the raw motor/asthenia ROR moved little and never changed rank: enzalutamide 3.32 to 3.42, apalutamide 1.72 to 1.81, darolutamide 2.81 to 2.79, abiraterone 1.44 to 1.56. Report-level duplication is unlikely to explain the raw signals. Re-running the full calibration and multiplicity correction on the duplicate-flag-removed data left the reading intact and, if anything, firmer: seven pairs kept a calibrated lower bound above one, none reached q < 0.05, and the smallest q rose from 0.088 to 0.190 (Supplementary Figure S4, Supplementary Table S5). Under the strongest deduplication openFDA supports, these signals still do not survive calibrated uncertainty.

### 2.8. Head-to-Head Comparison Against the Other ARPIs

**Figure 5:**
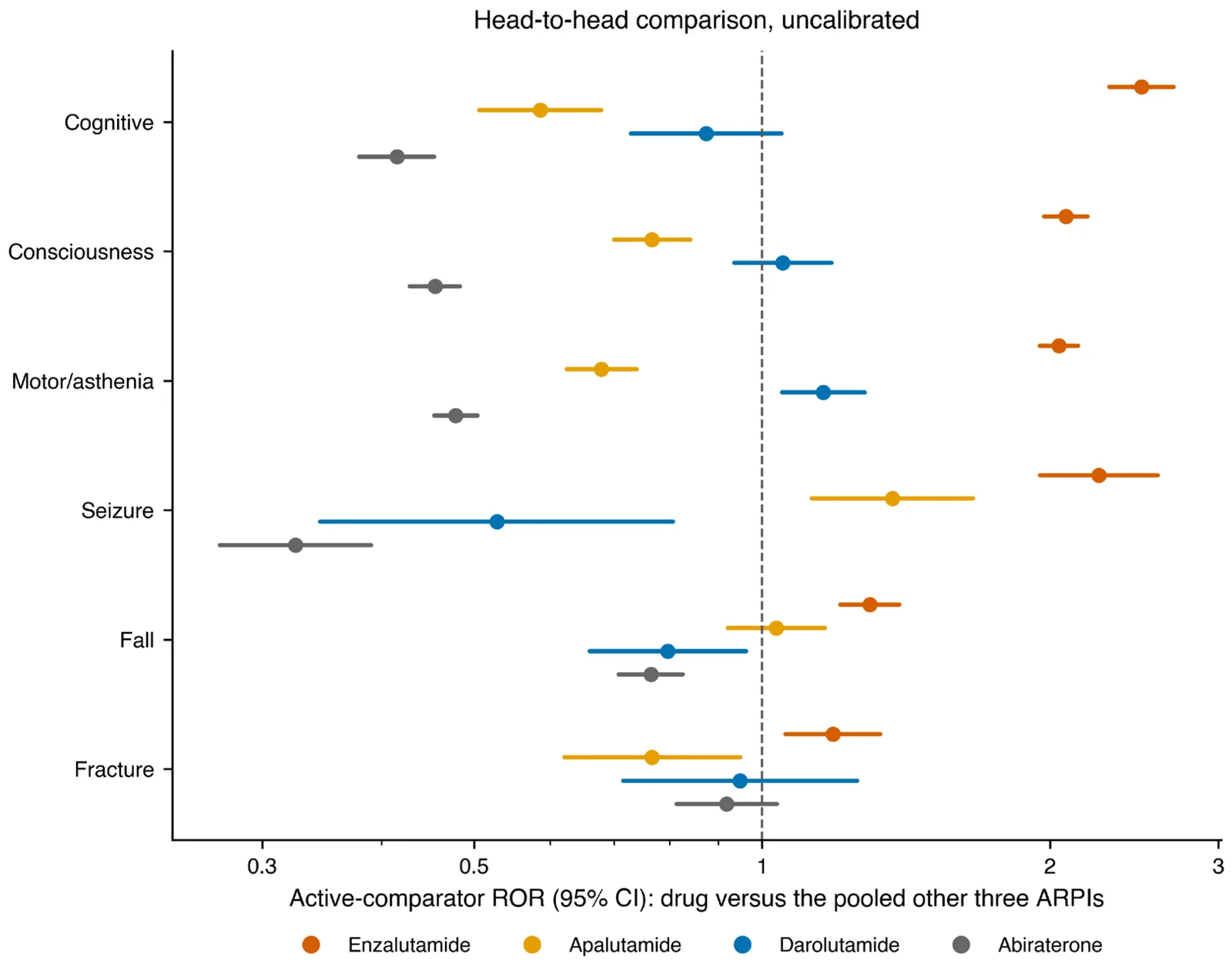
Head-to-head comparison against the pooled other three ARPIs. Active-comparator reporting odds ratios (drug versus the other three agents combined) with 95% confidence intervals, by event group. Enzalutamide exceeds the pooled comparator for every CNS group; abiraterone and apalutamide fall below one for most CNS groups because the comparator is dominated by enzalutamide, and darolutamide sits near one. Estimates are raw and share the prostate cancer indication but are not adjusted for inter-agent differences in reporting intensity.

**Figure 6:**
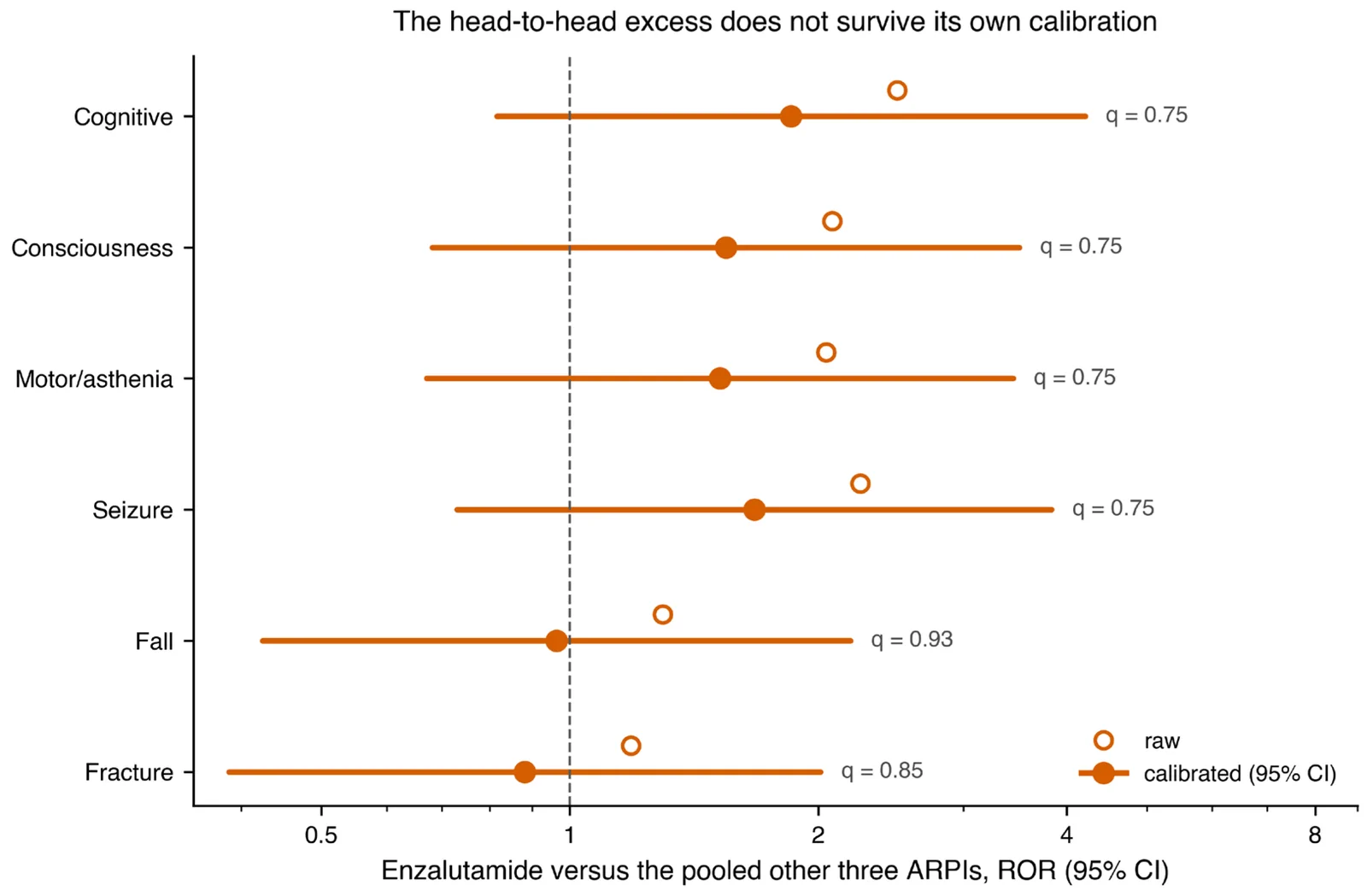
The head-to-head enzalutamide excess under empirical-null calibration. Enzalutamide against the pooled other three ARPIs by event group: open circles, raw reporting odds ratios; filled circles with 95% confidence intervals, estimates after calibration against a drug-versus-drug empirical null refitted from the 18 negative controls, with Benjamini–Hochberg correction across the 24 head-to-head tests and q annotated. The raw cognitive excess (2.49) falls to a calibrated 1.85 (0.82–4.21); no calibrated head-to-head signal keeps a lower bound above one and the minimum q is 0.75.

Against the whole database, every agent shares the indication of the reports it is compared with, so we repeated the analysis using the other three ARPIs as the comparator (Figure 5, Supplementary Table S6). On raw disproportionality, enzalutamide stood above the pooled other three for every CNS group—cognitive ROR 2.49 (95% CI 2.31–2.69), consciousness 2.08 (1.97–2.19), motor/asthenia 2.04 (1.95–2.14), seizure 2.25 (1.95–2.59)—and modestly for fall (1.30, 1.21–1.39) and fracture (1.19, 1.06–1.33). Abiraterone and apalutamide fell below one for most CNS groups because the pooled comparator is dominated by enzalutamide, and darolutamide sat near one. We then applied the same empirical-null calibration to this contrast, fitting a fresh null for each drug from the 18 negative controls computed against the pooled other three ARPIs. The head-to-head null was itself biased upward for enzalutamide (exp(μ) = 1.34): mechanistically inert events were reported about a third more often for enzalutamide than for the other agents, the inter-agent reporting bias made visible. After recalibration and Benjamini–Hochberg correction across the 24 head-to-head tests, enzalutamide’s headline cognitive excess fell to a calibrated ROR of 1.85 (95% CI 0.82–4.21; q = 0.75). No head-to-head CNS, fall, or fracture signal kept a lower bound above one or reached q < 0.05 (minimum q = 0.75; Figure 6, Supplementary Table S6).

About half of the raw head-to-head excess was the reporting bias the calibration removes. The head-to-head contrast locates the drug on which any CNS excess would concentrate, enzalutamide, but does not, on these data, establish a real pharmacological difference.

## 3. Discussion

The main result is that the comparative question is not resolvable with these data. In the largest FAERS dataset available, twenty-two raw signals became five after calibration, and under the conservative sample-standard-deviation fit of the null none survived multiplicity correction. Under the maximum-likelihood fit the framework itself prescribes, six survived, three of them CNS pairs. Both fits are defensible but not equivalent: the conservative sample-standard-deviation analysis and the framework-specified maximum-likelihood fit yielded different conclusions. The control-set resampling moves the count further still (Supplementary Figure S3), so the claim these data support is that the verdict is set by the calibration design rather than by the drugs. Either way, the CNS signals now used to separate these agents, which several published comparisons rest on [6,7], are too fragile to be a sound basis for choosing between them. A head-to-head comparison sharpens where any residual excess sits: on raw disproportionality the CNS excess is not spread across the AR antagonists but concentrates on enzalutamide (Figure 5). We then subjected that contrast to the same test as everything else. We calibrated the head-to-head estimates against a null refitted from the negative controls in the same drug-versus-drug frame. That null is itself biased upward for enzalutamide (exp(μ) = 1.34), the inter-agent reporting bias made explicit. After calibration and multiplicity correction, enzalutamide’s headline cognitive signal fell from 2.49 to 1.85 (95% CI 0.82–4.21), and no head-to-head signal survived (minimum q = 0.75; Figure 6). The most visible new number the raw method produces does not survive our method either. This does not exonerate enzalutamide (a real difference could be masked by the very bias that inflates the raw signal), but it means the head-to-head excess, like every other signal here, is not robust enough to stand alone in agent selection.

The negative result sits well with the better evidence. Randomized-trial meta-analyses put the ARPI-linked rise in falls and fractures near two-fold [3,4], and our raw fall RORs, 1.42 to 2.00 across the four agents, are of similar size, from a separate data stream. The likeness is reassuring but not a formal check: the trial estimate contrasts an ARPI added to androgen-deprivation therapy against that therapy alone, while the reporting ratio contrasts each drug against the whole database, so the two are not the same comparison. A network meta-analysis found no significant CNS association for darolutamide [5], and here the darolutamide motor signal also failed calibration, though its report volume explains as much of that as its pharmacology. Where we part from earlier work is method. A 13-year FAERS study read prominent nervous system signals for the AR antagonists straight from uncalibrated disproportionality [7]; we reproduce those raw numbers and then show they do not survive calibration and, under the conservative sample-standard-deviation fit, multiplicity correction; their survival under the framework’s maximum-likelihood fit is exactly the identifiability problem we report. To our knowledge, this is the first empirical-null calibration of an ARPI comparison, and it says such signals must not be read without one.

One caution belongs with any class comparison of these four agents: they share an indication, not a pharmacology. Abiraterone acetate inhibits CYP17A1 and is given with a corticosteroid, so its reports carry both the mineralocorticoid consequences of that mechanism and the effects of the co-administered steroid, which our positive controls show directly in its hypokalaemia and peripheral-edema signals. Enzalutamide, apalutamide, and darolutamide are direct androgen-receptor antagonists, and they differ among themselves in structure, blood–brain barrier penetration, half-life, and drug–drug interaction profile. Therapeutic interchangeability does not imply pharmacological equivalence or a comparable therapeutic index, and nothing in a disproportionality comparison can be read as establishing either.

Two readings look positive and are not. The first is the BBB gradient: darolutamide carries the stronger raw motor signal and fails calibration only because its small dataset gives the widest null, so no mechanism can be read from which agent’s signal survives. The second is the five survivors: four sat at calibrated *p* between 0.027 and 0.046 and fail multiplicity, and the one clearly significant pair, abiraterone death, is best explained by indication, since abiraterone treats more advanced, higher-mortality disease. That confounding is not abiraterone’s alone: all four drugs carry the advanced-cancer case-mix into their comparison with the mixed background, and the empirical null corrects each drug’s own reporting bias, not the shared indication. The within-report fall–fracture contrast, which holds the drug fixed, is the cleaner reading for that reason. Neither positive reading supports a central drug effect.

One methodological point stands on its own. The negative-control set is usually treated as a fixed background, but here the number of surviving signals ran from one to 16 as the null was refitted and the controls swapped. That is not a flaw to bury; it is the finding. Part of that range is mechanical: dropping to 12 of 18 controls thins the null and widens its scatter on its own, so the full-set estimators (five, eight, seven, and 11) are the fairer measure of control-set sensitivity. The resampled range shows how far the count can move once controls are lost. Even so, in a spontaneous-reporting comparison of co-indicated drugs, the count of signals is set by the calibration design about as much as by the drugs. The estimator of the null is the second such choice, and it moves the answer further than the control set does. Taking the null’s dispersion as the sample standard deviation of the control estimates absorbs the controls’ own sampling error, then adds it again through √(SE^2^ + σ^2^); that fit is conservative. The framework’s maximum-likelihood fit separates the two, and leaves six pairs significant. A comparative disproportionality study must therefore state, and test, how far its conclusions move with both the control set and the null estimator, which is rarely done.

One thing did hold. Fractures were enriched among fall reports 10- to 38-fold over a non-fall serious reference class. This is co-reporting rather than a proven sequence, and it belongs to no single agent, so we do not read it as a signal for one drug. Its worth is corroborative: it recovers, from an independent stream, the fall–fracture axis that trials already mark as the dominant serious harm of the class [3,4]. For an older population already losing bone to androgen deprivation, that convergence points one way. Falls and fractures remain the axis with the firmest evidence, which does not remove the need to watch cognitive endpoints.

Two messages follow for the clinic. The first is what not to do: do not choose between ARPIs on real-world CNS disproportionality. The cognitive and consciousness signals that dominate the naïve analysis, and much of the narrative around it, were gone at the calibration step, and the BBB-linked gradient was confounded with report volume. A move to darolutamide justified by those signals is not supported here. If a low-penetrance agent is chosen, the case for it must come from randomized data and from a patient’s own cognitive assessment, not from FAERS. Cognitive screening still belongs in frail patients, with tools such as mini-COG inside a G8- or Clinical Frailty Scale assessment [11]; it belongs to judging fitness, not to ranking agents.

The second message is where attention belongs: the skeleton. Adding an AR-pathway inhibitor to androgen-deprivation therapy significantly raises fracture and fall risk, roughly doubling it across randomized-trial meta-analyses [3,4]. Androgen deprivation raises it before any ARPI is added: in a cohort of 50,613 men, among those surviving at least five years, 19.4% of those on androgen-deprivation therapy sustained a fracture, against 12.6% of those not treated [12]. Guidelines already act on this. Current EAU-EANM-ESTRO-ESUR-ISUP-SIOG guidance calls for bone-density measurement by dual-energy X-ray absorptiometry (DEXA) at the start of long-term androgen-deprivation therapy, treats a T-score below −2.5, or below −1 with added risk factors such as a fall history, as high risk, and strongly recommends offering bone protection in combination treatment, with calcium and vitamin D, and makes a bone-protective agent mandatory only after an osteoporotic fracture [11]. PEACE-3 shows the scale of the benefit: mandatory bone-protecting agents cut one-year fractures from 15.6% to 2.6% in the enzalutamide arm, a six-fold fall [13]. Our data reinforce the same priority from the reporting side, where the one structure that held within the ARPI reports themselves was the fall–fracture link. When agents work equally well, fall-risk screening and bone protection are the firmer and more useful ground for comparing their safety.

### Limitations

Several limits bound the reading. Spontaneous reports cannot give causation or incidence: FAERS has no denominator, reports are selective and sometimes duplicated, and disproportionality measures reporting, not risk. Comparing agents adds the confounding of report volume, which we show decides which signals survive. The abiraterone death signal is most likely indication, not mechanism. Onset is a reporting-latency proxy. Calibration leans on the control set; the jackknife held and the set was cleaned by mechanism, but some misclassification may remain. The fall–fracture reference class controls for generic serious co-reporting, not for every within-report correlation. Two limits carry the most weight. We used openFDA’s report-level counts without a separate primaryid/caseid deduplication pass. Removing openFDA’s suspected-duplicate flag, the strongest deduplication it supports, left the calibrated and multiplicity-corrected result unchanged and the smallest q higher, not lower (Supplementary Table S5); a full case-level (caseid) build could still shift individual estimates, so the report-level analysis is primary and the deduplicated one is read as sensitivity. And the control set is consequential: the survivor count ran from one to 16 across resampling, so no single surviving pair is read as established. Three further limits belong here. The empirical null is assumed Gaussian on the log scale, a working model rather than a fitted law. The spread σ that widens every interval mixes each drug’s own reporting bias with heterogeneity between the control events, which differ in baseline reporting for reasons unrelated to any ARPI, so σ is best read as an upper bound on drug-specific uncertainty rather than a pure measure of it. And the head-to-head contrast of Section 2.8, though put through the same calibration, rests on a thin within-class control set; its collapse is read as a failure to establish a between-agent difference, not as proof that none exists.

Three limits bear directly on the central result. Our original analysis fitted the null with the sample standard deviation of the control estimates, which absorbs the controls’ own sampling error and is therefore conservative; we now report the framework’s maximum-likelihood fit beside it, and the two disagree. Neither is beyond question. At eighteen controls, σ is poorly determined, with a 95% profile-likelihood interval of 0.37 to 0.82 for enzalutamide and 0.13 to 0.50 for apalutamide, and the maximum-likelihood estimator is itself modestly biased downward at this sample size, so it is the less conservative of the two. The honest position is that σ is not pinned down well enough for either fit to settle the comparative question, and a larger and more carefully constructed control set is what would settle it.

## 4. Materials and Methods

### 4.1. Data Source

The data came from the openFDA distribution of FAERS (/drug/event endpoint), queried in June 2026 over the full database then indexed (N = 20,328,575 reports). Counts are at the report level. openFDA already resolves each safety report to its latest version, so no separate primaryid/caseid deduplication was run; duplicates were instead identified by openFDA’s own suspected-duplicate flag, which marks a report the FDA has linked to an earlier submission of the same case, and a sensitivity analysis below re-runs the full calibrated and multiplicity-corrected analysis with every flagged report removed. openFDA does not expose the case-level (caseid) key that a full case collapse would need, so that flag is the strongest deduplication the source supports. Drugs were matched on the harmonized patient.drug.openfda.generic_name field, which maps brand and generic names to the ingredient (Xtandi to enzalutamide, for example) and recovered far more reports than the raw medicinalproduct string. Events came from patient.reaction.reactionmeddrapt with exact-match term sets, and serious outcomes from the seriousnessdeath and seriousnesshospitalization flags. The primary analysis kept every report naming a drug. A sensitivity analysis kept only reports in which the drug was the suspect agent (drugcharacterization = 1) rather than a concomitant one. All queries, term lists, and field mappings sit in the Supplementary Materials archive for exact reproduction. The data are public and de-identified, so the study is not human-subjects research and did not require an ethics approval. Reporting follows the READUS-PV recommendations [14].

### 4.2. Drug and Event Definitions

The four ARPIs were matched on the harmonized generic_name field, with abiraterone and abiraterone acetate treated as one. Events were MedDRA Preferred Terms, grouped a priori. The CNS outcomes of interest covered cognition, consciousness, seizure, and motor/asthenia; the fall-centered group covered falls and fractures across 11 fracture terms; serious outcomes covered hospitalization and death. These last two are report-level seriousness flags, not reaction Preferred Terms, so their disproportionality reflects case-level seriousness reporting rather than a reaction-specific effect, and is read with that limit.

The negative-control set was built in two stages, and we describe both because the second stage was not pre-specified. The initial set held twelve common events chosen for frequency rather than mechanism: nausea, diarrhea, constipation, vomiting, rash, pruritus, dry skin, alopecia, hot flush, peripheral swelling, anemia, and hypertension. Fitting a null to that set put its mean above zero for three of the four drugs, and inspection showed why: five of the twelve carry an established on-label androgen-axis or ARPI effect. The set was therefore rebuilt under an explicit mechanism rule, admitting an event only when it has no plausible androgen-axis, CNS, or established ARPI mechanism. The rebuilt set holds eighteen events and none of the initial twelve survived into it: cataract, conjunctivitis, cystitis, dental caries, diverticulitis, epistaxis, gastrooesophageal reflux disease, gingival bleeding, hemorrhoids, hypersensitivity, influenza, inguinal hernia, nail disorder, nasopharyngitis, seasonal allergy, sinusitis, tinnitus, and toothache. Both sets are listed in Supplementary Table S7 and both are analyzed (Section 2.7). Because the rebuilt set was chosen after inspecting the first null, we call the resulting null the primary mechanism-filtered null rather than a pre-specified one, and we treat its dependence on that choice as a result rather than as a nuisance. The five events removed for carrying a true on-label effect are precisely what a positive control requires, and we use them as such (Section 4.7). The full MedDRA term dictionary for every group, including both control sets, is in the Supplementary Materials archive.

### 4.3. Disproportionality Metrics

Each drug–event pair gave a 2 × 2 table. From it we computed the reporting odds ratio (ROR) with a 95% confidence interval (CI), the proportional reporting ratio with a Yates-corrected χ^2^, and the information component with its 95% credible interval [15]. The reference for every primary estimate is the full FAERS background, so each ROR contrasts one drug against all other reports, not against another ARPI; a within-class comparator is taken up separately (Section 2.8).

### 4.4. Empirical-Null Calibration

Calibration used the negative controls as a measuring stick. For each drug, a Gaussian null N(μ, σ^2^) was fitted to the ln(ROR) values of the 18 controls, which by design have no true effect. Here, μ is the drug’s systematic reporting bias and σ the extra uncertainty that nominal confidence intervals miss [9,10]. Each estimate was then recalibrated as ln(ROR)_calibrated = ln(ROR)_observed − μ, with standard error √(SE^2^ + σ^2^).

The null can be fitted in two ways and the choice turns out to matter, so we report both. The first takes μ and σ as the unweighted mean and sample standard deviation of the individual control ln(ROR) values. This is the fit of our original analysis and it is the conservative one: the sample standard deviation of the observed control estimates contains both the systematic spread we want to measure and the controls’ own sampling error, which √(SE^2^ + σ^2^) then adds a second time. The second is the maximum-likelihood fit that the Schuemie framework specifies [10] and its reference implementation performs, in which each control contributes ln(ROR)_i_ ~ N(μ, σ^2^ + SE_i_^2^) so that the controls’ sampling error is separated from the systematic spread rather than absorbed into it. We report the sample-standard-deviation fit as a conservative bound and the maximum-likelihood fit as the framework’s own, and Section 2.3 gives the result under each.

The framework was built for confounding in longitudinal effect estimation. Carried to spontaneous reports, it treats each drug’s scatter across the control events as its reporting-bias distribution. The Gaussian form is a working model, not a fitted law.

### 4.5. Multiplicity and Robustness

Thirty-two pairs were tested, so the calibrated two-sided *p*-values were corrected across all of them with the Benjamini–Hochberg false-discovery-rate procedure; a signal counted as robust only at q below 0.05. Each null’s stability was checked by leave-one-out jackknife, refitting μ and σ with each control dropped in turn. To see why any signal lost significance, each raw-significant estimate was split two ways: the bias shift alone (subtracting μ, keeping the nominal standard error) and the full calibration (also adding σ to the standard error). To see how far the result depended on the controls, the null was refitted three more ways: an inverse-variance-weighted estimator, a minimum-count (≥5) subset, and 10,000 random 12-of-18 control subsets drawn under a fixed seed, each time recomputing the Benjamini–Hochberg q-values and recording both how many pairs survived calibration (lower bound above one) and how many reached q < 0.05. The 32 tests are not independent (the four drugs share one background, and fall and fracture co-report), so the false-discovery-rate procedure is read under its positive-dependence condition; with the smallest q at 0.088 under the primary null, the negative reading does not turn on the choice of correction.

We used the Benjamini–Hochberg procedure rather than Bonferroni because signal detection screens many hypotheses at once and tolerates a controlled proportion of false discoveries, and because it is the less conservative of the two, so that a negative finding under it is the stronger statement. In these data the distinction is moot: the smallest calibrated *p*-value was 0.00276, giving a Bonferroni-adjusted value of 0.088, identical to the smallest Benjamini–Hochberg q. Under the maximum-likelihood fit the choice is no longer immaterial. Of the six pairs that reach a Benjamini–Hochberg q below 0.05, four also clear a Bonferroni threshold; the two that do not are apalutamide with seizure and enzalutamide with motor/asthenia. The comparative CNS count therefore depends on the multiplicity procedure as well as on the null estimator.

As a complementary design that removes the shared prostate-cancer indication, we also computed active-comparator RORs: for each drug–event pair the index drug was contrasted against the pooled other three ARPIs rather than the whole database. These raw contrasts share indication but, like all spontaneous-report comparisons, do not adjust for differences in reporting intensity between agents (e.g., time on market).

### 4.6. Time-to-Onset and Fall–Fracture Co-Reporting Analyses

For reports with a suspect-drug start date, onset was taken as report date minus start date, capped at 0–1095 days. That window is a pragmatic bound rather than a modeled one: it excludes negative and implausibly long intervals, which in FAERS arise mostly from data-entry error, while spanning the period over which these agents are typically given. Because the analysis is exploratory and enters none of the conclusions, we did not tune it. FAERS carries no reaction-level event date, so this is a reporting-latency proxy, read only in comparison [16]. A two-parameter Weibull was fitted per drug and event. For the fall–fracture link, the share of fall reports also naming a fracture was measured against a reference class of non-fall serious reports for the same drug (a death or hospitalization flag, with falls excluded). The enrichment was tested with Fisher’s exact test. Sensitivity analyses were limited to suspect-drug reports. Analyses used Python 3 (requests, pandas, numpy, and scipy). The openFDA query strings, the analysis code, and the derived datasets are provided in the Supplementary Materials to permit full reproduction.

### 4.7. Validation: Positive Controls and the Detection Floor

A calibration that removes signals is only informative if it keeps the ones that are real. We therefore assembled positive controls from the four agents’ FDA labels, taking events with an established label-documented association with at least one agent and excluding any event that was a negative control or an outcome of interest. Twelve events qualified: hot flush, hypertension, rash, pruritus, anemia, hypokalaemia, hypothyroidism, gynaecomastia, weight decreased, arthralgia, peripheral edema, and alanine aminotransferase increased. Five of these are the events removed from the initial negative-control set, which is the reason they were removed. Each of the 48 drug–event pairs was put through the identical calibration, with Benjamini–Hochberg correction across the 48.

To express what the calibrated intervals can and cannot exclude, we also computed for each pair the smallest calibrated ROR detectable with 80% power at a two-sided α of 0.05, given that pair’s calibrated standard error. We report this detection floor alongside the observed estimates rather than describing any null result as an absence of effect.

## 5. Conclusions

Under the conservative sample-standard-deviation fit of the empirical null, no comparative ARPI CNS signal in FAERS survived calibration and multiplicity correction. Under the maximum-likelihood fit the same framework prescribes, six pairs did, three of them CNS pairs. Both are defensible, though the sample-standard-deviation fit is deliberately conservative and the maximum-likelihood fit more closely follows the framework, so the reading these data support is that the comparative CNS question is not identifiable from FAERS—not that it has been answered either way. What is firm is that the apparent statistical strength of the uncalibrated signals is overstated because the nominal uncertainty does not capture the additional reporting heterogeneity demonstrated by the negative controls, and that the calibrated intervals cannot exclude CNS differences in the magnitude at issue. FAERS CNS disproportionality should therefore not stand alone in choosing between these agents; that choice belongs to randomized and denominator-based evidence, to the agents’ differing pharmacology, and to an individual patient’s vulnerability. The fall and fracture risk of the class remains its dominant serious harm in trials and guidelines, and the within-report fall–fracture structure we find is consistent with that evidence without displacing attention from CNS effects.

## Figures and Tables

**Table 1:** Drug–event pairs keeping a calibrated 95% lower bound above one, with Benjamini–Hochberg false-discovery-rate (FDR) q-values across all 32 tests. No pair reaches q < 0.05 under this null.

Drug	Event	Calibrated ROR	95% CI	Calibrated *p*	FDR q
Enzalutamide	Motor/asthenia	4.43	1.17–16.8	0.029	0.26
Apalutamide	Motor/asthenia	2.98	1.07–8.27	0.036	0.26
Apalutamide	Fall	3.17	1.14–8.82	0.027	0.26
Abiraterone	Fall	3.09	1.02–9.32	0.046	0.26
Abiraterone	Death	5.39	1.79–16.3	0.003	0.088

ROR, reporting odds ratio; CI, confidence interval. Displayed *p*-values are rounded; q-values are computed on the unrounded vector of all 32 calibrated *p*-values with step-up monotonicity enforced. Death is a report-level seriousness flag rather than a reaction term, so the abiraterone–death pair reflects case-level seriousness reporting.

**Table 2:** Label-established positive controls under the same calibration, showing what the procedure retains and the level of its detection floor. Estimates are from the sample-standard-deviation fit of the null.

Positive Control	Agent	Raw ROR	Calibrated ROR (95% CI)	q
Hot flush	Enzalutamide	20.64	27.50 (7.26–104.24)	<0.001
Hot flush	Abiraterone	10.55	22.04 (7.30–66.50)	<0.001
Hypokalaemia	Abiraterone	8.61	17.98 (5.95–54.31)	<0.001
Rash	Apalutamide	5.82	10.10 (3.64–28.00)	<0.001
Hypothyroidism	Apalutamide	5.17	8.98 (3.17–25.38)	<0.001
Peripheral edema	Abiraterone	2.25	4.70 (1.55–14.22)	0.033
Alanine aminotransferase increased	Abiraterone	2.25	4.70 (1.55–14.26)	0.033
Gynaecomastia	Enzalutamide	2.45	3.26 (0.86–12.44)	0.235
Hypertension	Enzalutamide	1.24	1.65 (0.43–6.25)	0.617

ROR, reporting odds ratio; CI, confidence interval. q-values are Benjamini–Hochberg-corrected across all 48 positive-control pairs; the full set is in Supplementary Table S3. The last two rows are label-documented effects that did not survive calibration, and are shown to locate the detection floor. Hypokalaemia is specific to abiraterone (calibrated ROR 0.61–1.54 for the other three agents), hypothyroidism and rash to apalutamide (0.44–1.77 for the others).

## Data Availability

The data presented in this study are available in the openFDA distribution of the FDA Adverse Event Reporting System (FAERS) at https://open.fda.gov, accessed on 20 June 2026 a public-domain resource. All code, openFDA query strings, the MedDRA term dictionary, and the derived calibration, false-discovery-rate, and jackknife outputs are provided as a separate data archive with the Supplementary Materials; the openFDA extract is documented as a fixed June 2026 snapshot to permit full reproduction.

## Supplementary Materials

The following supporting information can be downloaded at: https://www.mdpi.com/article/10.3390/ph19081213/s1. Figures S1–S4: robustness analyses; Weibull time-to-onset; stability of the multiplicity verdict; deduplication sensitivity. Tables S1–S7: case demographics; the full 32-pair disproportionality; all 48 drug–event pairs from the twelve positive-control events; fall–fracture co-reporting counts; the duplicate-flag-removed 32-pair disproportionality; active-comparator reporting odds ratios; and the two negative-control sets. A separate data archive contains the analysis code, the openFDA query strings, the MedDRA term dictionary, and every derived dataset, including the positive-control, maximum-likelihood-null, resampling and bootstrap outputs added in this revision.
