# Supplementary material for: Empirical-Null Calibration Challenges Comparative CNS Safety Signals for Androgen-Receptor Pathway Inhibitors: A FAERS Re-Analysis
Source: Pharmaceuticals (Basel). 2026 Aug 1;19(8):1213. doi: 10.3390/ph19081213 (PMC13516075; doi:10.3390/ph19081213)
Supplement: Supplementary file 1 [file pharmaceuticals-19-01213-s001.zip › pharmaceuticals-4451093-supplementary.pdf]

pharmaceuticals

MDPI

Supplementary Materials

Supplementary Figures

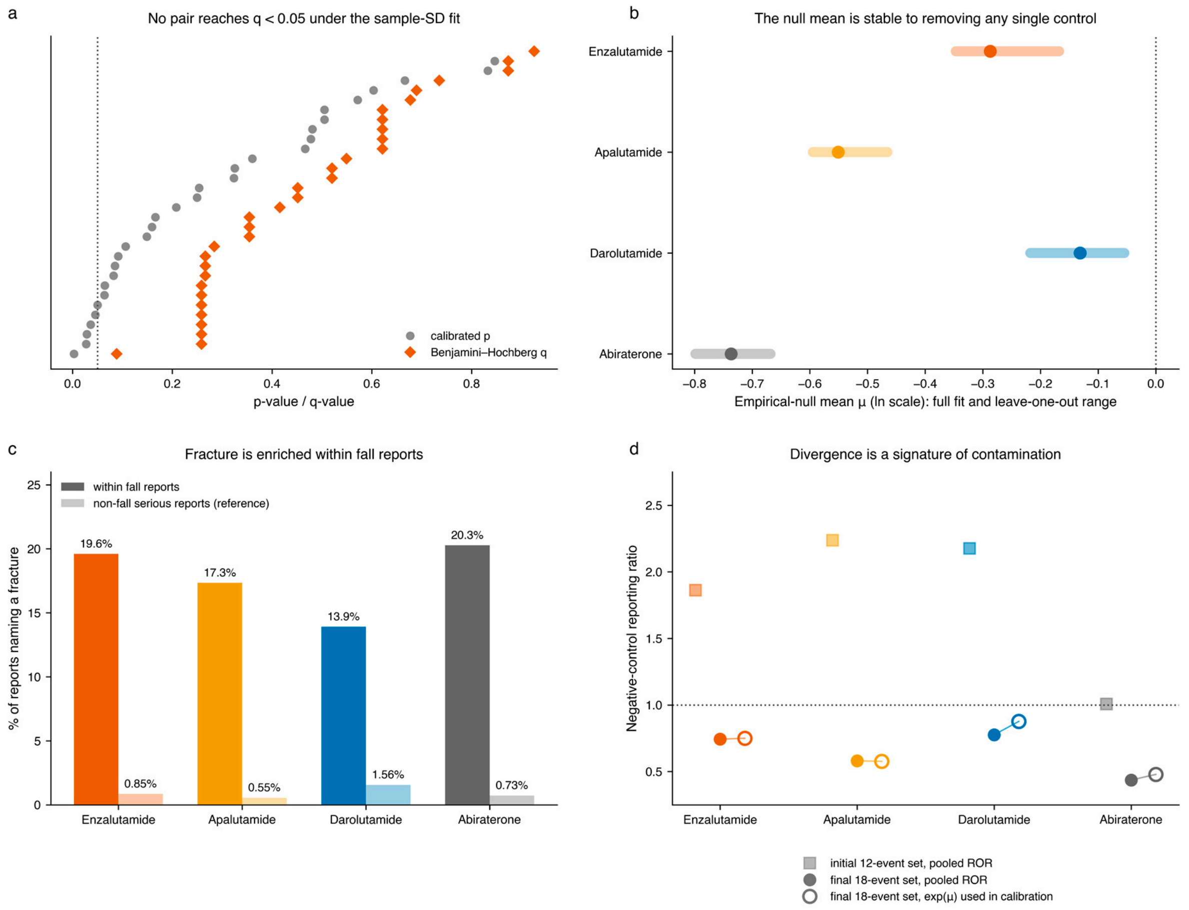

Supplementary Figure S1. Robustness analyses. (a) Calibrated p-values versus Benjamini–Hochberg FDR q across all 32 tests: no signal reaches q<0.05. (b) Leave-one-out jackknife of the empirical-null mean μ: stable to removal of any single negative control. (c) Fracture co-reporting within fall reports versus a non-fall serious-report reference class (10–38× enrichment). (d) On the final 18-event control set the pooled 2×2 ROR and the exp(μ) used in calibration agree, both below 1; the initial 12-event set, which still held five on-label effects, pools above 1. The divergence is a signature of contamination, not of skewed control counts.

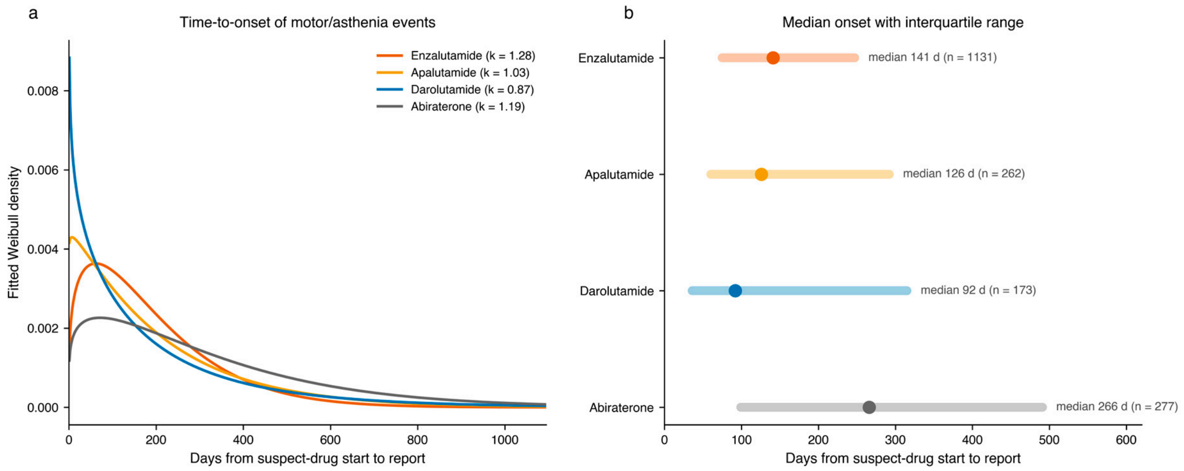

Supplementary Figure S2. Weibull time-to-onset of motor/asthenia events by agent. (a) Fitted onset densities; (b) median onset with interquartile range. Onset does not order by BBB penetration. This analysis is exploratory (FAERS lacks reaction-level event dates) and supports no pharmacokinetic interpretation.

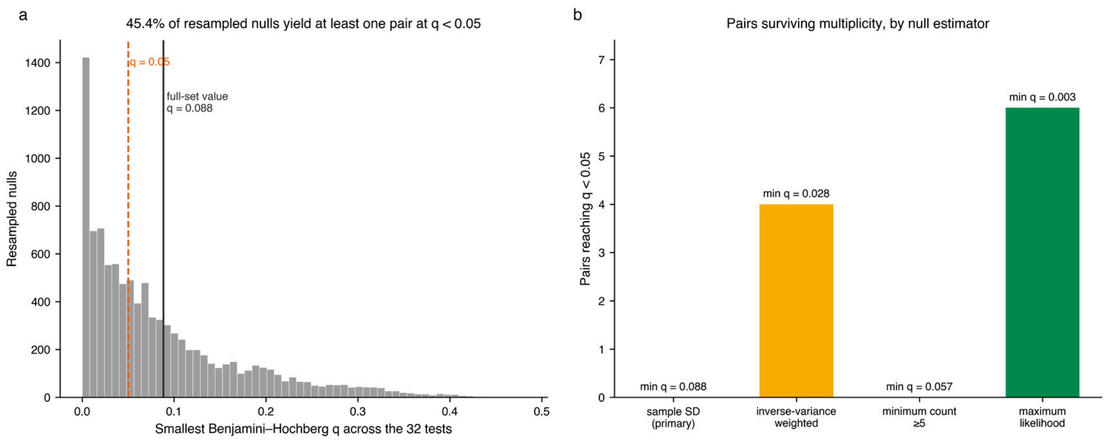

Supplementary Figure S3. Stability of the multiplicity verdict to the calibration design. (a) Distribution of the smallest Benjamini–Hochberg q across the 32 tests over 10,000 random 12-of-18 negative-control subsets; the dashed line marks q = 0.05 and the solid line the primary full-set value (q = 0.088). Forty-five percent of resampled nulls yield at least one pair at q < 0.05. (b) Pairs surviving at q < 0.05 under four full-set null estimators: the primary sample-standard-deviation null (0), an inverse-variance-weighted null (4), a minimum-count (≥5) subset (0), and the maximum-likelihood null (6); the smallest q is annotated above each bar.

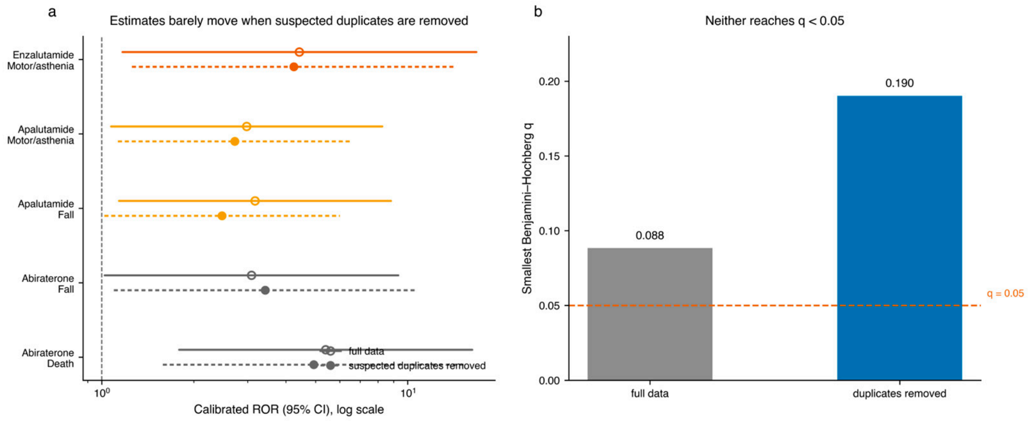

Supplementary Figure S4. Deduplication sensitivity. (a) Calibrated ROR (95% CI) for the pairs that keep a lower bound above 1, on the full data (open circles) versus after removing openFDA's suspected-duplicate flag (filled circles); the estimates barely move. (b) Smallest Benjamini–Hochberg q across the 32 tests: 0.088 on the full data and 0.190 after duplicate-flag removal. Neither reaches q < 0.05.

Supplementary Table S1. Reported case demographics by agent.

Drug	Reports	Sex recorded, n (%)	Male, % of recorded	Female, n	Age recorded, %	Median age, y (IQR)
Enzalutamide	58,003	55,739 (96.1%)	99.2	363	57.5	76 (69–82)
Apalutamide	11,281	9,604 (85.1%)	99.6	40	51.3	76 (70–82)
Darolutamide	5,120	4,287 (83.7%)	99.6	19	60.3	74 (66–80)
Abiraterone	40,471	37,858 (93.5%)	99.3	217	55.0	75 (68–81)

Sex and age are as captured in FAERS; the sex and age percentages are of reports with that field populated. Cases are almost entirely male and elderly, consistent with the advanced prostate cancer indication. The percentages are of reports with the field populated; "Sex recorded" includes reports coded with unknown or unspecified sex, so the male and female percentages need not sum to 100.

Supplementary Table S2. Full background-referenced disproportionality for all 32 drug–event pairs (four ARPIs × eight event groups), raw and after empirical-null calibration, with Benjamini–Hochberg FDR q.

Drug	Event	Group	Raw ROR (95% CI)	Calibrated ROR (95% CI)	p (calibrated)	q (FDR)
Enzalutamide	Cognition	CNS	1.95 (1.87–2.04)	2.61 (0.69–9.88)	0.159	0.355
Apalutamide	Cognition	CNS	0.83 (0.72–0.96)	1.45 (0.52–4.04)	0.481	0.621
Darolutamide	Cognition	CNS	1.20 (1.00–1.43)	1.37 (0.33–5.68)	0.666	0.735
Abiraterone	Cognition	CNS	0.71 (0.66–0.78)	1.49 (0.49–4.51)	0.478	0.621
Enzalutamide	Consciousness	CNS	1.92 (1.87–1.98)	2.56 (0.68–9.71)	0.166	0.355
Apalutamide	Consciousness	CNS	1.11 (1.02–1.21)	1.93 (0.69–5.36)	0.208	0.416
Darolutamide	Consciousness	CNS	1.48 (1.32–1.67)	1.69 (0.41–6.98)	0.466	0.621
Abiraterone	Consciousness	CNS	0.80 (0.76–0.85)	1.67 (0.55–5.05)	0.360	0.549
Enzalutamide	Seizure	CNS	1.47 (1.36–1.59)	1.96 (0.52–7.44)	0.323	0.520
Apalutamide	Seizure	CNS	1.40 (1.17–1.68)	2.44 (0.87–6.85)	0.091	0.266
Darolutamide	Seizure	CNS	0.57 (0.38–0.87)	0.65 (0.15–2.85)	0.571	0.677
Abiraterone	Seizure	CNS	0.45 (0.38–0.54)	0.95 (0.31–2.89)	0.925	0.925
Enzalutamide	Motor/asthenia	CNS	3.32 (3.23–3.41)	4.43 (1.17–16.77) †	0.029	0.258
Apalutamide	Motor/asthenia	CNS	1.72 (1.58–1.86)	2.98 (1.07–8.27) †	0.036	0.258
Darolutamide	Motor/asthenia	CNS	2.81 (2.55–3.10)	3.21 (0.78–13.21)	0.106	0.284
Abiraterone	Motor/asthenia	CNS	1.44 (1.38–1.51)	3.02 (1.00–9.10)	0.050	0.258
Enzalutamide	Fall	Fall–fracture	2.00 (1.91–2.10)	2.67 (0.70–10.12)	0.149	0.355
Apalutamide	Fall	Fall–fracture	1.83 (1.64–2.04)	3.17 (1.14–8.82) †	0.027	0.258
Darolutamide	Fall	Fall–fracture	1.42 (1.18–1.71)	1.62 (0.39–6.75)	0.505	0.621
Abiraterone	Fall	Fall–fracture	1.48 (1.39–1.58)	3.09 (1.02–9.32) †	0.046	0.258
Enzalutamide	Fracture	Fall–fracture	1.47 (1.36–1.58)	1.95 (0.51–7.42)	0.325	0.520
Apalutamide	Fracture	Fall–fracture	1.06 (0.86–1.30)	1.84 (0.65–5.20)	0.250	0.451
Darolutamide	Fracture	Fall–fracture	1.28 (0.97–1.69)	1.46 (0.35–6.17)	0.603	0.690
Abiraterone	Fracture	Fall–fracture	1.28 (1.16–1.41)	2.67 (0.88–8.08)	0.082	0.266
Enzalutamide	Hospitalization	Serious	0.86 (0.84–0.87)	1.14 (0.30–4.32)	0.846	0.874
Apalutamide	Hospitalization	Serious	1.04 (1.00–1.09)	1.81 (0.65–5.01)	0.254	0.451
Darolutamide	Hospitalization	Serious	1.02 (0.96–1.09)	1.16 (0.28–4.79)	0.832	0.874

Abiraterone	Hospitalization	Serious	1.36 (1.33–1.39)	2.84 (0.94–8.55)	0.064	0.258
Enzalutamide	Death	Serious	2.42 (2.37–2.47)	3.22 (0.85–12.21)	0.085	0.266
Apalutamide	Death	Serious	1.51 (1.43–1.59)	2.61 (0.94–7.25)	0.065	0.258
Darolutamide	Death	Serious	1.42 (1.31–1.54)	1.62 (0.39–6.66)	0.505	0.621
Abiraterone	Death	Serious	2.58 (2.52–2.64)	5.39 (1.79–16.26) †	0.003	0.088

† marks the five pairs that kept a calibrated 95% lower bound above 1. Raw significance (ROR 95% lower bound > 1) held for 22 pairs; none reached q < 0.05 (smallest q = 0.088, abiraterone–death). The abiraterone motor/asthenia lower bound is 0.9997 and is shown rounded to 1.00; it does not exceed 1, which is why that pair carries no dagger. Event groups: cognition, consciousness, seizure, and motor/asthenia (CNS outcomes of interest); fall and fracture; hospitalization and death (serious outcomes).

Supplementary Table S3. All 48 label-established positive-control pairs, raw and after the same empirical-null calibration, with Benjamini–Hochberg q across the 48.

Positive control	Drug	Reports	Raw ROR	Calibrated ROR (95% CI)	q
Alanine Aminotransferase Increased	Abiraterone	266	2.25	4.70 (1.55–14.26)	0.033
Alanine Aminotransferase Increased	Apalutamide	20	0.60	1.04 (0.34–3.17)	0.938
Alanine Aminotransferase Increased	Darolutamide	41	2.74	3.12 (0.74–13.26)	0.280
Alanine Aminotransferase Increased	Enzalutamide	117	0.68	0.91 (0.24–3.50)	0.925
Anaemia	Abiraterone	700	1.91	3.99 (1.32–12.06)	0.062
Anaemia	Apalutamide	125	1.21	2.11 (0.75–5.92)	0.344
Anaemia	Darolutamide	87	1.87	2.14 (0.51–8.91)	0.495
Anaemia	Enzalutamide	861	1.64	2.18 (0.57–8.27)	0.465
Arthralgia	Abiraterone	574	0.71	1.49 (0.49–4.51)	0.620
Arthralgia	Apalutamide	338	1.54	2.66 (0.96–7.42)	0.194
Arthralgia	Darolutamide	219	2.22	2.53 (0.61–10.47)	0.382
Arthralgia	Enzalutamide	2137	1.91	2.54 (0.67–9.63)	0.355
Gynaecomastia	Abiraterone	32	0.52	1.09 (0.34–3.46)	0.925
Gynaecomastia	Apalutamide	32	1.87	3.25 (1.11–9.53)	0.117
Gynaecomastia	Darolutamide	15	1.94	2.21 (0.49–9.90)	0.495
Gynaecomastia	Enzalutamide	214	2.45	3.26 (0.86–12.44)	0.235
Hot Flush	Abiraterone	1465	10.55	22.04 (7.30–66.50)	<0.001
Hot Flush	Apalutamide	846	22.61	39.22 (14.14–108.81)	<0.001

Hot Flush	Darolutamide	583	35.72	40.73 (9.90–167.66)	<0.001
Hot Flush	Enzalutamide	3850	20.64	27.50 (7.26–104.24)	<0.001
Hypertension	Abiraterone	785	1.93	4.04 (1.34–12.21)	0.062
Hypertension	Apalutamide	216	1.91	3.31 (1.18–9.23)	0.090
Hypertension	Darolutamide	53	1.02	1.16 (0.28–4.90)	0.925
Hypertension	Enzalutamide	725	1.24	1.65 (0.43–6.25)	0.617
Hypokalaemia	Abiraterone	740	8.61	17.98 (5.95–54.31)	<0.001
Hypokalaemia	Apalutamide	22	0.89	1.54 (0.51–4.64)	0.603
Hypokalaemia	Darolutamide	6	0.53	0.61 (0.12–3.09)	0.694
Hypokalaemia	Enzalutamide	120	0.94	1.26 (0.33–4.82)	0.864
Hypothyroidism	Abiraterone	16	0.26	0.54 (0.16–1.81)	0.495
Hypothyroidism	Apalutamide	88	5.17	8.98 (3.17–25.38)	<0.001
Hypothyroidism	Darolutamide	3	0.38	0.44 (0.07–2.68)	0.559
Hypothyroidism	Enzalutamide	38	0.43	0.57 (0.15–2.25)	0.603
Oedema Peripheral	Abiraterone	530	2.25	4.70 (1.55–14.22)	0.033
Oedema Peripheral	Apalutamide	101	1.53	2.65 (0.94–7.48)	0.195
Oedema Peripheral	Darolutamide	56	1.87	2.13 (0.51–8.98)	0.495
Oedema Peripheral	Enzalutamide	507	1.49	1.99 (0.52–7.56)	0.495
Pruritus	Abiraterone	165	0.23	0.47 (0.15–1.44)	0.372
Pruritus	Apalutamide	270	1.35	2.35 (0.84–6.55)	0.258
Pruritus	Darolutamide	73	0.80	0.91 (0.22–3.81)	0.925
Pruritus	Enzalutamide	578	0.56	0.74 (0.19–2.81)	0.790
Rash	Abiraterone	341	0.40	0.84 (0.28–2.55)	0.870
Rash	Apalutamide	1229	5.82	10.10 (3.64–28.00)	<0.001
Rash	Darolutamide	162	1.55	1.77 (0.43–7.33)	0.603
Rash	Enzalutamide	834	0.69	0.92 (0.24–3.50)	0.925
Weight Decreased	Abiraterone	619	1.15	2.40 (0.79–7.26)	0.280
Weight Decreased	Apalutamide	236	1.58	2.74 (0.98–7.66)	0.184
Weight Decreased	Darolutamide	86	1.26	1.44 (0.35–6.02)	0.757
Weight Decreased	Enzalutamide	1779	2.35	3.13 (0.83–11.88)	0.248

Estimates use the sample-standard-deviation fit of the null. Positive controls were taken from the four agents' FDA labels; none is a negative control or an outcome of interest. Under this fit 13 pairs keep a calibrated lower bound above 1 and 9 reach q < 0.05; under the maximum-likelihood fit the corresponding counts are 20 and 18.

Supplementary Table S4. Within-report fall–fracture co-reporting by agent.

Drug	Fall reports	Fracture in fall, n (%)	Non-fall serious reports	Fracture in non-fall, n (%)	Enrichment OR	Fisher p
Enzalutamide	1811	355 (19.6%)	19,543	167 (0.85%)	28.3	1.5×10^{-260}
Apalutamide	323	56 (17.3%)	3608	20 (0.55%)	37.6	4.2×10^{-46}
Darolutamide	115	16 (13.9%)	1599	25 (1.56%)	10.2	1.5×10^{-9}
Abiraterone	942	191 (20.3%)	17,413	127 (0.73%)	34.6	2.2×10^{-166}

Columns, per ARPI: fall reports; fall reports also naming a fracture, n (%); non-fall serious reports (a death or hospitalization flag, with falls excluded); those naming a fracture, n (%); the enrichment odds ratio; and the Fisher exact p. The enrichment odds ratio runs from 10.2 (darolutamide) to 37.6 (apalutamide), the 10- to 38-fold odds quoted in the text, and is distinct from the percentage ratio (fall% ÷ non-fall%), which runs lower.

Supplementary Table S5. Full 32-pair disproportionality after removing openFDA's suspected-duplicate flag, raw and after empirical-null calibration, with Benjamini–Hochberg FDR q.

Drug	Event	Raw ROR (95% CI)	Calibrated ROR (95% CI)	p (calibrated)	q (FDR)
Enzalutamide	Cognition	1.76 (1.60–1.95)	2.19 (0.65–7.43)	0.208	0.417
Apalutamide	Cognition	0.88 (0.75–1.03)	1.32 (0.54–3.23)	0.536	0.780
Darolutamide	Cognition	1.30 (1.07–1.58)	1.23 (0.29–5.25)	0.778	0.830
Abiraterone	Cognition	0.76 (0.67–0.85)	1.54 (0.49–4.82)	0.461	0.737
Enzalutamide	Consciousness	1.79 (1.67–1.92)	2.22 (0.66–7.51)	0.200	0.417
Apalutamide	Consciousness	1.00 (0.91–1.12)	1.51 (0.63–3.65)	0.357	0.635
Darolutamide	Consciousness	1.45 (1.27–1.65)	1.38 (0.33–5.82)	0.665	0.819
Abiraterone	Consciousness	0.83 (0.77–0.90)	1.68 (0.54–5.26)	0.370	0.635
Enzalutamide	Seizure	2.70 (2.34–3.13)	3.36 (0.98–11.44)	0.053	0.212
Apalutamide	Seizure	1.73 (1.40–2.14)	2.61 (1.06–6.42) †	0.037	0.212
Darolutamide	Seizure	0.68 (0.41–1.11)	0.64 (0.14–2.93)	0.568	0.780
Abiraterone	Seizure	0.52 (0.40–0.67)	1.05 (0.33–3.38)	0.932	0.932
Enzalutamide	Motor/asthenia	3.42 (3.22–3.63)	4.24 (1.25–14.36) †	0.020	0.212
Apalutamide	Motor/asthenia	1.81 (1.65–1.98)	2.72 (1.13–6.57) †	0.026	0.212
Darolutamide	Motor/asthenia	2.79 (2.50–3.12)	2.65 (0.63–11.18)	0.185	0.417
Abiraterone	Motor/asthenia	1.56 (1.46–1.67)	3.17 (1.01–9.88) †	0.047	0.212
Enzalutamide	Fall	2.18 (1.97–2.42)	2.71 (0.80–9.20)	0.110	0.296
Apalutamide	Fall	1.64 (1.44–1.88)	2.47 (1.02–6.00) †	0.045	0.212
Darolutamide	Fall	1.38 (1.11–1.71)	1.31 (0.31–5.58)	0.718	0.830
Abiraterone	Fall	1.69 (1.54–1.85)	3.42 (1.09–10.70) †	0.034	0.212
Enzalutamide	Fracture	1.94 (1.67–2.27)	2.41 (0.71–8.24)	0.159	0.392
Apalutamide	Fracture	1.00 (0.78–1.28)	1.51 (0.61–3.74)	0.377	0.635
Darolutamide	Fracture	1.31 (0.95–1.81)	1.24 (0.29–5.42)	0.771	0.830
Abiraterone	Fracture	1.28 (1.10–1.49)	2.59 (0.82–8.15)	0.104	0.296
Enzalutamide	Hospitalization	0.97 (0.93–1.02)	1.21 (0.36–4.08)	0.762	0.830
Apalutamide	Hospitalization	0.91 (0.86–0.96)	1.36 (0.57–3.28)	0.488	0.744
Darolutamide	Hospitalization	0.93 (0.86–1.00)	0.88 (0.21–3.70)	0.859	0.887
Abiraterone	Hospitalization	1.24 (1.20–1.28)	2.52 (0.81–7.85)	0.111	0.296
Enzalutamide	Death	1.06 (1.00–1.13)	1.32 (0.39–4.47)	0.655	0.819
Apalutamide	Death	1.54 (1.44–1.64)	2.32 (0.96–5.57)	0.061	0.216

Darolutamide	Death	1.57 (1.43–1.73)	1.49 (0.35–6.30)	0.585	0.780
Abiraterone	Death	2.43 (2.34–2.52)	4.93 (1.58–15.37) †	0.006	0.190

† marks the seven pairs that kept a calibrated 95% lower bound above 1; none reaches q < 0.05 (smallest q = 0.190). Compare Supplementary Table S2 (full data).

Supplementary Table S6. Active-comparator reporting odds ratios (each ARPI versus the pooled other three), by event group, raw and after empirical-null calibration with Benjamini–Hochberg correction across the 24 head-to-head tests.

Drug	Event group	Reports (drug/others)	Raw ROR	Calibrated ROR (95% CI)	q
Abiraterone	Cognition	595/2585	0.41	0.66 (0.31–1.37)	0.75
Apalutamide	Cognition	193/2987	0.59	0.63 (0.24–1.61)	0.75
Darolutamide	Cognition	125/3055	0.87	0.66 (0.26–1.66)	0.75
Enzalutamide	Cognition	2267/913	2.49	1.85 (0.82–4.21)	0.75
Abiraterone	Consciousness	1366/5305	0.46	0.72 (0.34–1.50)	0.75
Apalutamide	Consciousness	521/6150	0.77	0.82 (0.32–2.09)	0.82
Darolutamide	Consciousness	311/6360	1.05	0.80 (0.32–1.97)	0.82
Enzalutamide	Consciousness	4473/2198	2.08	1.55 (0.68–3.50)	0.75
Abiraterone	Motor/asthenia	1893/6925	0.48	0.76 (0.36–1.58)	0.75
Apalutamide	Motor/asthenia	622/8196	0.68	0.72 (0.28–1.85)	0.75
Darolutamide	Motor/asthenia	447/8371	1.16	0.88 (0.36–2.17)	0.85
Enzalutamide	Motor/asthenia	5856/2962	2.04	1.52 (0.67–3.44)	0.75
Abiraterone	Seizure	138/774	0.33	0.52 (0.24–1.10)	0.75
Apalutamide	Seizure	118/794	1.37	1.46 (0.56–3.79)	0.75
Darolutamide	Seizure	22/890	0.53	0.40 (0.15–1.08)	0.75
Enzalutamide	Seizure	634/278	2.25	1.67 (0.73–3.83)	0.75
Abiraterone	Fall	942/2249	0.77	1.21 (0.58–2.53)	0.82
Apalutamide	Fall	323/2868	1.03	1.10 (0.43–2.83)	0.87
Darolutamide	Fall	115/3076	0.80	0.60 (0.24–1.51)	0.75
Enzalutamide	Fall	1811/1380	1.30	0.96 (0.42–2.19)	0.93
Abiraterone	Fracture	401/802	0.92	1.45 (0.69–3.06)	0.75
Apalutamide	Fracture	93/1110	0.77	0.82 (0.31–2.13)	0.82
Darolutamide	Fracture	51/1152	0.95	0.72 (0.28–1.84)	0.75
Enzalutamide	Fracture	658/545	1.19	0.88 (0.39–2.01)	0.85

Counts are index-drug / pooled-comparator reports with the event. The calibration null is refitted for each drug from the 18 negative controls in the drug-versus-drug frame.

Supplementary Table S7. The two negative-control sets. The initial set was chosen for frequency; the final set was rebuilt under a mechanism rule after five of the initial events were found to carry established on-label effects.

#	Initial 12-event set (superseded)	Final 18-event set (used)
1	Nausea	Cataract
2	Diarrhoea	Conjunctivitis
3	Constipation	Cystitis
4	Vomiting	Dental Caries
5	Rash †	Diverticulitis
6	Pruritus †	Epistaxis
7	Dry Skin	Gastrooesophageal Reflux Disease
8	Alopecia	Gingival Bleeding

9	Hot Flush †	Haemorrhoids
10	Peripheral Swelling	Hypersensitivity
11	Anaemia †	Influenza
12	Hypertension †	Inguinal Hernia
13	—	Nail Disorder
14	—	Nasopharyngitis
15	—	Seasonal Allergy
16	—	Sinusitis
17	—	Tinnitus
18	—	Toothache

† marks the five events with an established on-label androgen-axis or ARPI effect; these were removed from the control set and are used instead as positive controls (Section 4.7, Table 2). No event of the initial set is retained in the final set. Pooled 2×2 reporting odds ratios (enzalutamide / apalutamide / darolutamide / abiraterone): initial set 1.86 / 2.24 / 2.18 / 1.01; final set 0.74 / 0.58 / 0.78 / 0.44.
